# Safe and Early Primary Closure of Open Abdomen in Pediatric Liver Transplantation Using a Doppler‐Guided Tension Relief Strategy and Enhanced Wound Environment

**DOI:** 10.1155/joot/9317796

**Published:** 2026-05-31

**Authors:** Michael Gurevich, Tomer Mendelson, Haya Fischer, Elhanan Nahum, Avichai Weissbach, Yael Glassberg Mozer, Orith Waisbourd-Zinman, Sigal Aizner, Eviatar Nesher, Moris Topaz

**Affiliations:** ^1^ Faculty of Medicine, Tel Aviv University, Tel Aviv, Israel, tau.ac.il; ^2^ Liver Transplantation Unit, Schneider Children’s Medical Center, Petah Tikva, Israel, schneider.org.il; ^3^ Urology Department, Tel Aviv Sourasky Medical Center, Tel Aviv, Israel, tau.ac.il; ^4^ Department of Pediatrics, Mayanei Hayeshua Medical Center, Bnei Brak, Israel, mymc.co.il; ^5^ Pediatric Critical Care Unit, Schneider Children’s Medical Center, Petah Tikva, Israel, schneider.org.il; ^6^ Institute of Gastroenterology, Nutrition, and Liver Diseases, Schneider Children’s Medical Center, Petah Tikva, Israel, schneider.org.il; ^7^ Organ Transplant Department, Rabin Medical Center, Beilinson Hospital, Petah Tikva, Israel, clalit.co.il

## Abstract

Pediatric liver transplantation (LT) with large‐for‐size (LFS) grafts frequently necessitates open abdomen (OA) management due to donor–recipient graft size mismatch, increasing the risk of delayed closure and frequently requires the use of permanent prosthetic materials, together compounding the risk of postoperative infection. This study evaluates the safety and effectiveness of the Topaz–Gurevich Doppler‐guided controlled‐closure technique (DGCT) in facilitating early primary abdominal closure without graft size reduction or the use of permanent prosthetic implants. A retrospective review was conducted at Schneider Children’s Medical Center (2016–2024), including 21 pediatric LT recipients requiring OA management, primarily due to LFS grafts. DGCT integrates a tension relief system (TRS) for gradual abdominal wall approximation, guided by real‐time Doppler ultrasound to monitor perfusion and modulate closure tension, and is complemented by regulated, oxygen‐enriched irrigation and negative pressure–assisted wound therapy to optimize the local wound environment. All patients achieved primary abdominal closure within a median of 8 days (range, 2–23); only the first three cases required the temporary use of prosthetic materials. The median PICU and hospital stays were 17 and 35 days, respectively. No cases of graft failure, retransplantation, or mortality occurred. One patient developed sepsis related to a bowel leak and fully recovered. DGCT proved safe and effective in managing OA in pediatric LT, avoiding permanent prosthetic implantation, and enabling early closure while maintaining graft perfusion. Its combined approach of mechanical tension control and enhanced local wound conditions reduces the need for graft size reduction, minimizes infection risk, and may broaden the donor pool. Based on high‐risk cases involving immunosuppressed children with significant graft–recipient mismatch, these findings support DGCT as a valuable strategy for pediatric transplantation. Moreover, the principles demonstrated here may be extrapolated to the closure of complex OA scenarios across broader pediatric and adult surgical populations.

## 1. Introduction

Pediatric liver transplantation (LT) complicated by the need for open abdomen (OA) management remains a major surgical and physiological challenge. Early abdominal closure is strongly desirable in this population due to immunosuppression, fluid and heat loss, increased infection risk, and prolonged ventilator dependence. However, excessive closure tension can precipitate intra‐abdominal hypertension and abdominal compartment syndrome, leading to compromised graft perfusion, respiratory failure, and multiorgan dysfunction. One of the principal drivers for OA in pediatric LT is a high graft‐to‐recipient weight ratio (GRWR).

Because of the limited availability of size‐matched deceased donors, pediatric LT frequently relies on split or partial grafts from living or deceased donors, an approach that has proven safe and effective for expanding the donor pool [1]. Nevertheless, even reduced‐size grafts may exceed the abdominal domain in small children, resulting in so‐called large‐for‐size (LFS) transplantation, commonly defined as a GRWR of approximately ≥ 2.5–3.5%. LFS grafts are associated with an increased risk of abdominal compartment syndrome, hemodynamic instability, respiratory compromise, vascular inflow and outflow obstruction, graft ischemia, and early graft failure. These risks are further exacerbated by bowel and hepatic congestion following portal vein reperfusion and the frequent need for high‐volume fluid resuscitation in the immediate perioperative period [2–5]. Despite these well‐recognized hazards, the ongoing shortage of pediatric liver grafts often necessitates acceptance of LFS grafts, particularly in urgent or life‐saving transplant scenarios.

To address the inability to achieve safe primary closure (PC), multiple strategies for OA management and delayed abdominal closure have been described. Prosthetic materials, most commonly synthetic meshes, have been used to bridge fascial defects and facilitate staged closure. Alternative materials and adjuncts, including polytetrafluoroethylene (ePTFE; Gore‐Tex) mesh, biologic meshes (including porcine‐derived matrices), abdominal fascia allografts, and tissue expanders, have also been reported, reflecting the absence of a universally accepted standard technique [6–10]. While these approaches can facilitate abdominal closure, they are associated with important limitations, most notably an increased risk of prosthetic infection in immunosuppressed pediatric transplant recipients, as highlighted by recent data, showing that early bacterial infection, including wound and intra‐abdominal sources, is a major driver of morbidity and mortality after pediatric LT [11–13].

In addition, delayed‐closure strategies frequently require multiple reoperations for graft size reduction or prosthesis removal, and, in some cases, result in permanent abdominal wall patches. Permanent prosthetic materials may impair ultrasound imaging and complicate percutaneous liver biopsy, particularly via anterior epigastric approaches. Some centers maintain prolonged antibiotic therapy until prosthesis removal, followed by secondary intention skin closure, which may adversely affect functional and aesthetic outcomes [6]. Absorbable prostheses, while avoiding permanent foreign material, may predispose to progressive fascial retraction and increase the risk of subsequent ventral hernia formation [7]. Collectively, these limitations underscore the need for alternative closure strategies that minimize prosthetic use while maintaining physiologic safety.

In this context, we present a retrospective analysis of the Topaz–Gurevich Doppler‐guided controlled‐closure technique (DGCT), a novel approach designed to achieve early‐delayed abdominal closure without the use of permanent prosthetic materials in pediatric LFS LT. DGCT integrates a tension relief system (TRS) that enables controlled, staged abdominal wall approximation under continuous physiologic feedback, guided by real‐time Doppler ultrasonography of hepatic perfusion and concurrent intra‐abdominal pressure (IAP) monitoring. This is combined with regulated, oxygen‐enriched irrigation and negative pressure–assisted wound therapy (ROINPT) to optimize the wound environment and facilitate safe closure. Accordingly, we evaluated the feasibility, safety, and clinical relevance of DGCT as a physiologically guided, prosthesis‐sparing strategy for OA management in pediatric LT when immediate PC is contraindicated by size mismatch and/or the risk of intra‐abdominal hypertension with compromised graft perfusion and anticipated or evolving abdominal compartment physiology.

## 2. Materials and Methods

### 2.1. Study Design and Patient Selection

We conducted a retrospective cohort study of all patients < 18 years of age who underwent LT requiring OA management at Schneider Children’s Medical Center (SCMC), Israel, between July 2016 and June 2024. Consecutive OA cases in which the DGCT was employed during the perioperative period were included. All included patients had a minimum follow‐up of 11 months. During the study period, all OA cases were managed using DGCT; therefore, no control group treated with alternative closure techniques was available. No exclusion criteria were applied beyond failure to meet the predefined inclusion criteria. The primary endpoint was time to primary abdominal closure. Secondary endpoints included ICU length of stay, hospital length of stay, mortality, graft failure or retransplantation, and perioperative and late complications. Data were extracted from electronic medical records.

### 2.2. Data Collection and Comparative Analysis

Collected data included patient demographics, clinical diagnoses, surgical history, graft characteristics, operative techniques, complications, Doppler ultrasound findings, postoperative courses, and outcomes. Because there was no control group, we compared ICU and hospital lengths of stay and mortality rates between DGCT‐treated patients and liver transplant recipients without OAs during the same period, who served as the reference group. Medians, interquartile range (IQRs), and ranges were calculated, and differences between medians were analyzed using the Mann–Whitney test. Age, weight, and group (DGCT‐treated patients and liver transplant recipients without OAs) were analyzed together using multivariable linear regression with log‐transformed outcomes.

### 2.3. Ethics Approval and Informed Consent

This retrospective observational study was approved by the Institutional Review Board (IRB number 0550‐22‐RMC). All patients’ legal guardians had previously signed institutional surgical informed consent forms that included permission for anonymized use of clinical data for research. In accordance with local IRB policy and national guidelines for retrospective minimal‐risk research, additional study‐specific informed consent was waived and is therefore not applicable.

### 2.4. DGCT Technique Overview

The DGCT strategy combines the TopClosure^®^ TRS and ROINPT via Vcare α^®^ systems and wound dressing kits (IVT Medical Ltd., Ra’anana, Israel), as previously described [14, 15]. The TRS consists of a set of plastic polymer attachment plates linked by approximation straps designed to facilitate the gradual, reversible closure of wound edges. These plates are strategically positioned along the wound margins, approximately 2–3 cm from the edges. For neonates with particularly sensitive skin, placing the plates on hydrocolloid dressing adhesive sheaths (HAS) (Granuflex^®^, ConvaTec, Flintshire, United Kingdom) is recommended to protect the delicate skin surface and increase the area of attachment of the TRS to the skin. The number of TRS sets required depends on the wound size and the expected tension across the wound. The approximation straps between the plates enable stepwise edge approximation via a lock‐release mechanism, facilitating primary wound closure without skin undermining. Instead, DGCT promotes *en bloc* incremental stretching of the skin and the underlying abdominal muscles through mechanical creep, bridging the wound while minimizing tension and dead space and related complications.

### 2.5. Objective Criteria for OA, Indication Within the DGCT Protocol

The DGCT strategy was applied to manage temporary OA through staged, progressive approximation in predefined clinical scenarios, including inability to achieve primary fascial and skin approximation at the initial operation; intentional OA when abdominal compartment syndrome was anticipated due to the use of a LFS graft or marked bowel congestion; and situations in which a planned re‐exploration (“second‐look” procedure) was anticipated with a high likelihood of abdominal reopening. In this framework, temporary OA is an integral component of DGCT rather than a failure of closure, enabling controlled, reversible, and physiologically guided approximation of the abdominal wall.

Clinical indicators prompting temporary OA within DGCT include excessive tension during attempted fascial approximation and/or any demonstrable compromise of hepatic arterial or portal venous inflow. Decisions are guided by objective physiologic parameters, including IAP targets of ≤ 10–12 mmHg and Doppler‐derived perfusion metrics, with particular emphasis on maintaining portal vein velocity ≥ 40 cm/s, assessed before and during each approximation attempt. IAP is continuously monitored via an indwelling urinary catheter, while hepatic perfusion is evaluated using intraoperative and postoperative Doppler ultrasonography. Exceeding predefined IAP thresholds or deterioration in portal venous flow mandates continued temporary OA, with staged, reversible DGCT‐guided closure rather than forced definitive closure. The lock‐release mechanism of the TopClosure^®^ allows immediate adjustment or reversal of approximation, preserving physiologic safety while permitting controlled progression toward definitive closure.

### 2.6. Application of Covering ROINPT Dressing System

Following approximation of the wound edges using the TRS, the wound is insulated from the external environment using ROINPT via the Vcare α^®^ system. A temporary sterile polyethylene (PE) generic plastic sheet (sterile organ bag) is fenestrated with 5‐ to 10‐mm slits made using an 11‐blade, placed over the internal organs, and spread to cover the flanks to prevent their adhesion to the abdominal wall and reduce cocoon formation. The Vcare^®^ system dressing consists of an off‐white, open‐cell, soft polyurethane (PU) sponge with an airtight, laminated PU upper layer. This design allows clear visualization of wound bleeding and local infection through the staining of the covering sponge. The laminated first‐layer sponge is fenestrated too, using a surgical blade, similar to the fenestration of the underlying plastic sheet, and is applied with its laminated surface facing the abdominal cavity. This configuration provides an additional barrier that prevents direct contact between the intra‐abdominal organs and the open‐cell sponge, as well as between the TopClosure^®^ straps and the underlying tissues. An additional sponge layer is placed above the TRS to serve as a top cover, allowing the straps to glide and facilitate further tissue approximation when necessary. A recent modification includes creating an opening in the sponge directly over the transplanted liver, forming an ultrasound‐transparent window that allows continuous postoperative monitoring of arterial and portal flow (Supporting video, File S1).

### 2.7. Oxygen and Vacuum Regulation

The entire dressing system is sealed with an adhesive drape. Nanofiltered oxygen is delivered through a distal port at 1–3 L/min, maintaining an atmospheric pO_2_ of 60%–80% to inhibit anaerobic bacterial proliferation. The Vcare^®^ system is set to operate at a low, oscillating negative pressure (40–60 mmHg), designed to reduce the risk of bleeding and internal organ injury, and to minimize granulation tissue growth.

### 2.8. Staged Closure Protocol

Progressive approximation of the TRS plates can be achieved without dismantling the dressing by temporarily suspending suction and adjusting strap tension at the bedside. As intra‐abdominal congestion subsides, staged closure is completed either in the pediatric intensive care unit (PICU) or the operating theater.

After complete abdominal closure, ROINPT is maintained for several days, while TRS remains in place for several weeks to support wound edge protection and minimize tension.

### 2.9. Supporting Information

A Supporting video (File S1) demonstrating the core surgical technique is provided separately to visually support the methodology and implementation of DGCT in pediatric LT.

## 3. Results

### 3.1. Patient Demographics and Clinical Indications

Between July 2016 and June 2024, 129 pediatric patients underwent LT at SCMC, all of whom had at least 11 months of follow‐up. An OA approach was required in 21 (16.3%) of these patients due to conditions that prevented primary abdominal closure during their perioperative course. These 21 patients were managed with the DGCT, while the remaining 108 patients served as the reference group.

In the reference group, the median age at LT was 3 years and 6 months (IQR 14 months to 9 years and 1 month, range 3 months to 18 years and 5 months), with a median body weight of 14.3 kg (IQR 8.7 to 28.1 kg; range, 5.7–80.0 kg; data missing in two patients). Among the DGCT group, 13 (62%) patients were male, with a median age at LT of 15 months (IQR 9 months to 2 years and 5 months; range, 4 months to 15 years and 9 months) (Table 1). The median body weight was 8.2 kg (IQR: 7.2–11.0 kg; range, 4.4–43.8 kg), and 18 (85.7%) weighed 11 kg or less. Differences observed in age and weight between the study group and reference group were significant (*p* = 0.003 and *p* = 0.001, respectively).

**TABLE 1:** Baseline demographics and clinical profile.

Case	Gender	Age at LT years (y) months (m)	Weight (kg)	Diagnosis—indication for LT	Type of LT	Previous operations
1	F	1 y 7 m	8.2	BA	LDLT	Kasai
2	F	1 y 8 m	8.75	PA	LDLT	—
3	M	3 y 5 m	11	PH1	DDLT	—
4	M	11 y 9 m	27.5	PFIC	DDLT	—
5	M	0 y 5 m	7.4	BA	LDLT	Kasai
6	M	1 y 0 m	8	OTCD	DDLT	—
7	M	1 y 6 m	9.2	BA	LDLT	Kasai
8	M	2 y 2 m	11	BA, FHF	LDLT	Kasai, DDLT
9	M	0 y 4 m	4.5	FHF	LDLT	—
10	M	0 y 5 m	7	FHF	LDLT	—
11	M	1 y 2 m	8.1	BA	DDLT	Kasai
12	M	0 y 9 m	6.3	BA	DDLT	—
13	M	15 y 9 m	43.8	ARPKD	DDLT	—
14	F	0 y 5 m	4.35	BA	LDLT	Kasai
15	F	4 y 7 m	13.6	ARPKD	DDLT	—
16	M	2 y 8 m	10.9	FHF	LDLT	—
17	M	1 y 3 m	10.1	BA	DDLT	Kasai
18	F	1 y 8 m	8.0	PH1	DDLT	_
19	F	0 y 9 m	6.8	BA	DDLT	Kasai
20	F	0 y 9 m	7.4	BA	DDLT	Kasai
21	F	0 y 11 m	9.4	BA	LDLT	Kasai

*Note:* PH1, primary hyperoxaluria type 1; OTCD, ornithine transcarbamylase deficiency.

Abbreviations: BA, biliary atresia; DDLT, deceased donor liver transplantation; FHF, fulminant hepatic failure; LDLT, living donor liver transplantation; LT, liver transplantation; PA, propionic acidemia; PFIC, progressive familial intrahepatic cholestasis.

### 3.2. Transplant Details and Surgical Characteristics

Biliary atresia was the most common indication for LT, occurring in 11 (52.4%) patients. Among these, 10 (90.9%) had undergone Kasai portoenterostomy before LT, and one had a prior LT. Other indications included fulminant hepatic failure, propionic acidemia, primary hyperoxaluria Type 1, progressive familial intrahepatic cholestasis, ornithine transcarbamylase deficiency, and autosomal recessive polycystic kidney disease. All 10 patients who received living donor LT (LDLT) were transplanted with left lateral segment (LLS) liver grafts (segments 2–3). The remaining 11 underwent deceased donor LT (DDLT), including 6 with split grafts and 3 with whole liver grafts.

In 12 (57%) of the 21 patients, the GRWR was 3.5% or greater; in 7 patients, it ranged from 2.5% to 3.5% (Table 2).

**TABLE 2:** Surgical and postoperative details with clinical findings.

Case	Graft type	GRWR (%)	Abdomen status at the end of transplantation	Transition to OA (POD)	Indication for OA	TGM start (POD)	Additional materials
1	LLS	4.5	OA + Bogota Bag	—	ET	4	Permacol™ Mesh
2	LLS	2.5	OA + Bogota Bag	—	ET	18	PTFE Mesh
3	LLS	3.8	PC	6	IAF	7	Vicryl^®^ Mesh
4	LLS	1.9	PC	4	IPF	4	—
5	Reduced LLS	5.6	OA + TGM	—	IPF	0	—
6	LLS	3.7	OA + TGM	—	IAF	0	—
7	LLS	4.2	PC	1	IAF	1	—
8	LLS	2.7	OA + TGM	—	ET	0	—
9	Reduced LLS	5.1	OA + TGM	—	ET	0	—
10	LLS	2.8	OA + TGM	—	ET	0	—
11	LLS	4.9	PC	3	IPF	3	—
12	Whole liver	3.5	OA + TGM	—	IPF	0	—
13	Whole liver	2.8	PC	2	IAF	5	—
14	LLS	4.3	OA + TGM	—	ET	0	—
15	Whole liver	2.7	PC	2	IAF	2	—
16	LLS	2.0	PC	7	ET	7	—
17	LLS	4.1	OA + TGM	—	ET	0	—
18	Whole liver	3.1	PC	2	IAF	2	—
19	LLS Cadaver	3.7	OA + TGM	—	ET	0	—
20	LLS	2.7	OA + TGM	—	ET	0	—
21	LLS	4.3	OA + TGM	—	ET	0	—

*Note:* DGCT, Topaz–Gurevich Doppler‐guided controlled‐closure technique.

Abbreviations: ET, excessive tension; GRWR, graft‐to‐recipient weight ratios; IAF, impaired arterial flow; IPF, impaired portal flow; LLS, left lateral segment; OA, open abdomen; PC, primary closure.

### 3.3. OA Indications and Management

In 11 patients, the indication for OA management was excessive closure tension, while in 10 patients, it was vascular compromise, evidenced by impaired arterial or portal venous flow (Table 2). Abdomens of thirteen patients remained open at the end of their LT.

• The three initial cases: In the first patient, DGCT was applied several days postoperatively following initial OA management with a Bogota bag. A Permacol™ biomesh implant was still required to bridge a 4‐cm fascial gap. In the second patient, a PTFE sheet was initially placed prior to DGCT but was later completely removed due to Candida infection. Subsequently, the abdominal wall was sufficiently stretched using the TopClosure^®^ system to achieve fascial closure. In the third patient, PC was achieved, but delayed OA was required due to impaired arterial blood flow. This patient achieved primary fascial closure buttressed by an absorbable Vicryl mesh. These were the only patients in whom permanent or absorbable implants were initially used. No permanent implants remained in place in this cohort in the long term.
• Subsequent cases: In 7 additional patients, PC was initially achieved but later required further interventions, including OA management. These patients ultimately underwent successful delayed PC with DGCT.
• Later cases: After gaining confidence in the DGCT approach, it was employed immediately at the end of surgery in the remaining 11 patients, eliminating the need for permanent or absorbable prostheses.

### 3.4. Infectious Complications and Outcomes

Routine abdominal fluid cultures were obtained to monitor for infectious complications in patients with OA. Of the 21 patients, 12 had sterile fluid cultures throughout their treatment. Anastomotic bowel or bile leaks were observed and repaired in 6 patients. Three of 21 patients (14%) developed biliary or intestinal leaks following DGCT application. One of these patients developed sepsis, which was successfully managed with antibiotic therapy and wound care. Three other patients had positive cultures of unknown origin, which resolved with sensitivity‐directed antibiotics. DGCT was not suspended in any patient due to infections. A total of six reoperations (29%) were required, mainly for source control.

### 3.5. Primary Outcomes and Long‐Term Follow‐Up

• Median time to primary abdominal closure: 8 days (range: 2–23 days).
• Median Vcare α^®^ dressing duration: 14 days (range: 4–34 days).
• Median TopClosure^®^ system duration: 17 days (range: 4–28 days).

Over a median follow‐up period of 38 months (range, 6–95 months), no deaths or additional liver transplants occurred. One patient (Case 1, Figure 1) developed a late incisional site seroma necessitating the Permacol™ removal, after which primary fascial closure was achieved. No late infections or hernias were observed.

FIGURE 1Case 1: First experience with Topaz–Gurevich Doppler‐guided controlled‐closure technique (DGCT) in pediatric liver transplantation. A 1‐year, 7‐month‐old girl with biliary atresia underwent LDLT (graft‐to‐recipient weight ratio 3.8%), requiring OA management due to graft size and intestinal distension. (a) At the end of transplantation, a Bogota bag, Kerlix^®^ dressing, and sterile drape were applied to close the muscular–fascial layer. (b) On POD 2, three TopClosure^®^ tension relief systems (TRS) sets (black arrows) were placed bedside to facilitate gradual vector‐oriented abdominal wall approximation and prevent muscular retraction. (c) Doppler US‐guided PICU bedside approximation session. (d) On POD 4, six TRS sets were applied and approximated under Doppler guidance in the operating room (OR), with Vcare α® vacuum dressings replacing the Bogota bag. (e, f) On POD 8, reoperation due to fever revealed that fascial closure was still unachievable. A Permacol™ prosthesis was used to bridge midline and transverse defects, followed by full‐thickness skin closure. Regulated, oxygen‐enriched irrigation and negative pressure–assisted wound therapy (ROINPT) was applied to minimize tension and promote healing. (g) On POD 14, TRS and ROINPT were removed. At six months, an incisional site seroma required drainage and removal of the Permacol™ implant. (h) The patient experienced no infections, maintained normal liver graft function, and achieved excellent functional and cosmetic outcomes.

**Figure:**
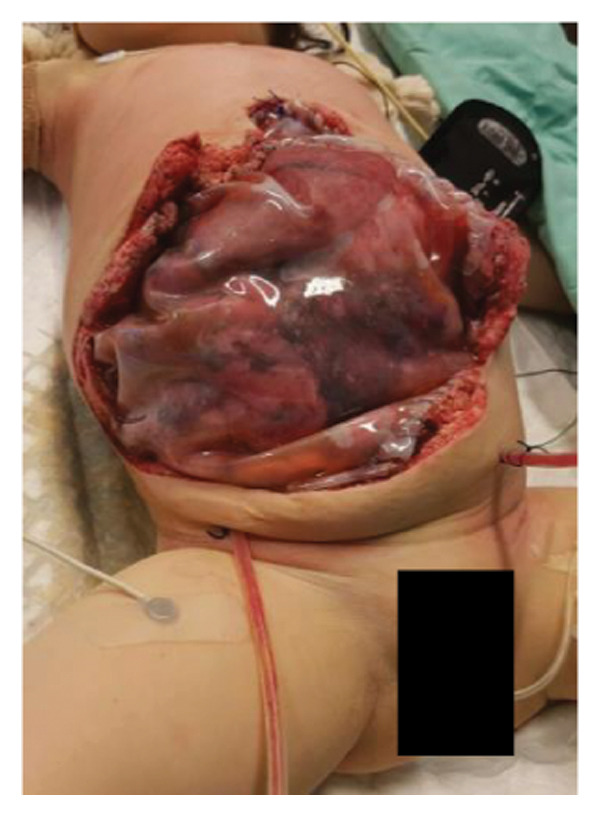
(a)

**Figure:**
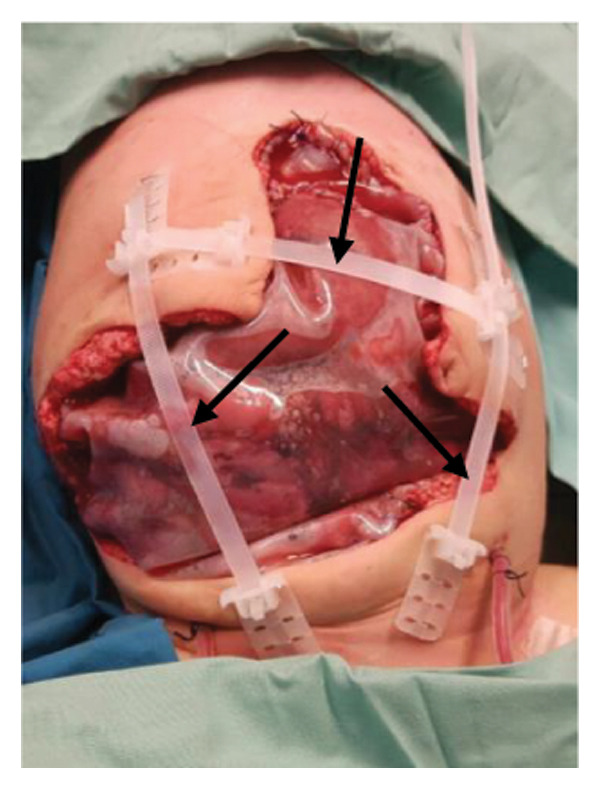
(b)

**Figure:**
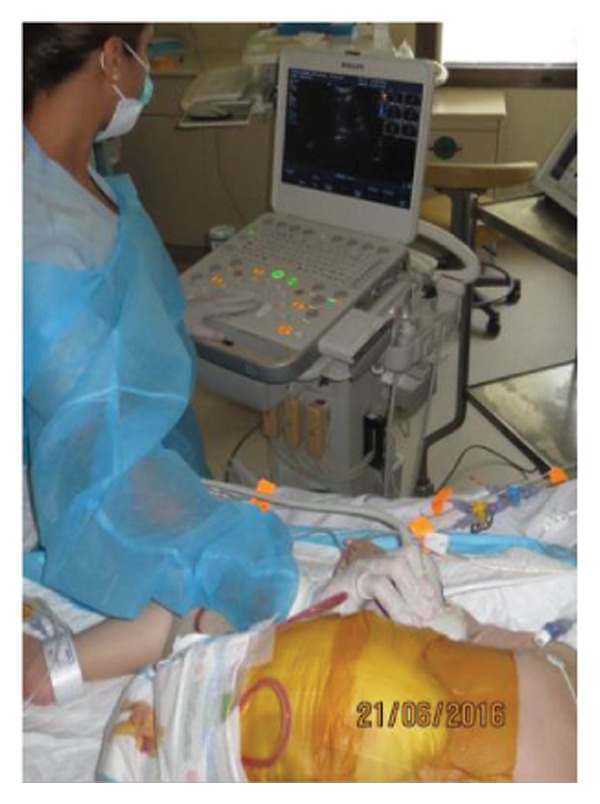
(c)

**Figure:**
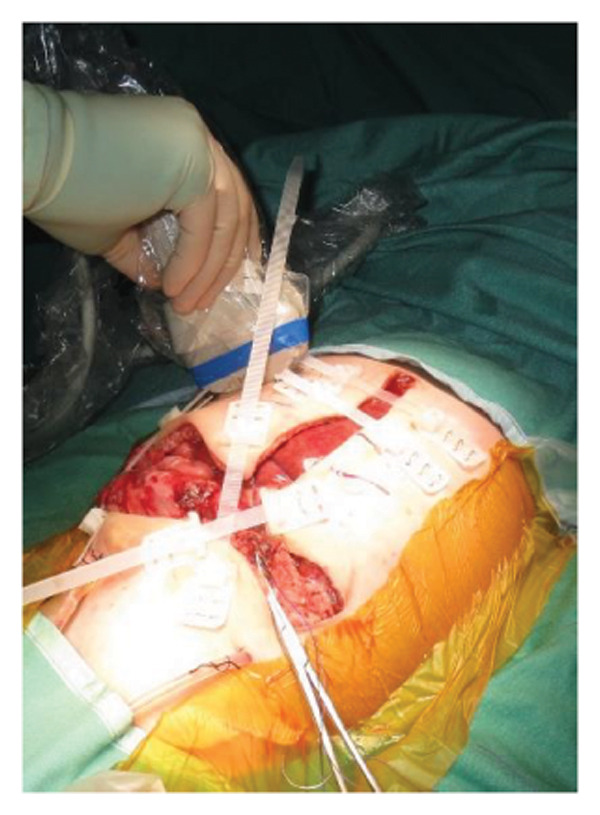
(d)

**Figure:**
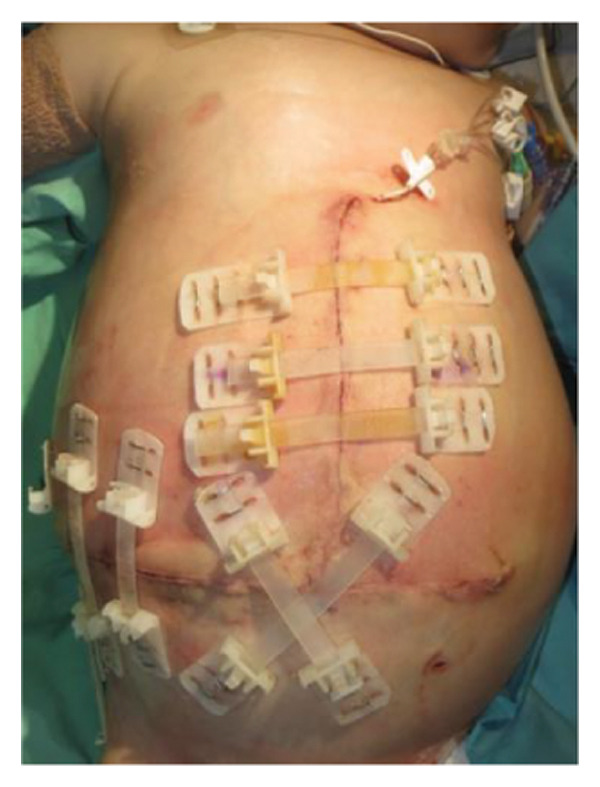
(e)

**Figure:**
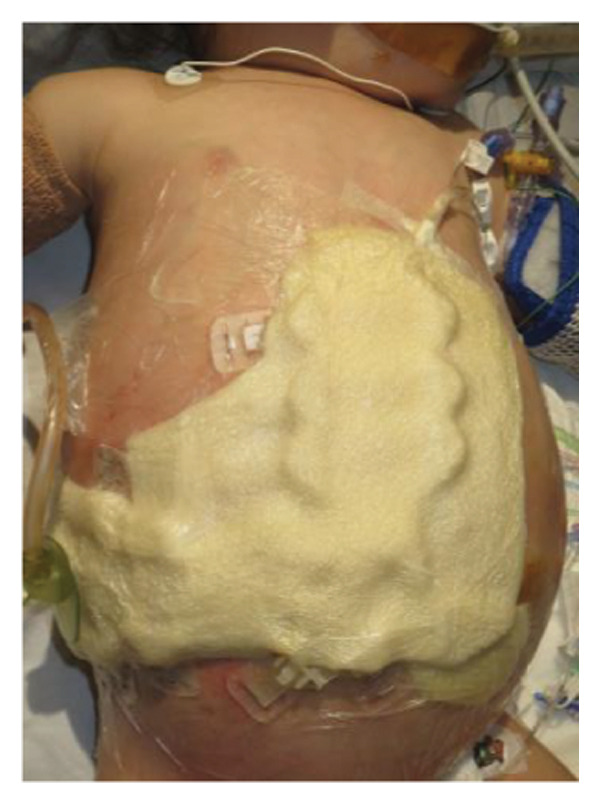
(f)

**Figure:**
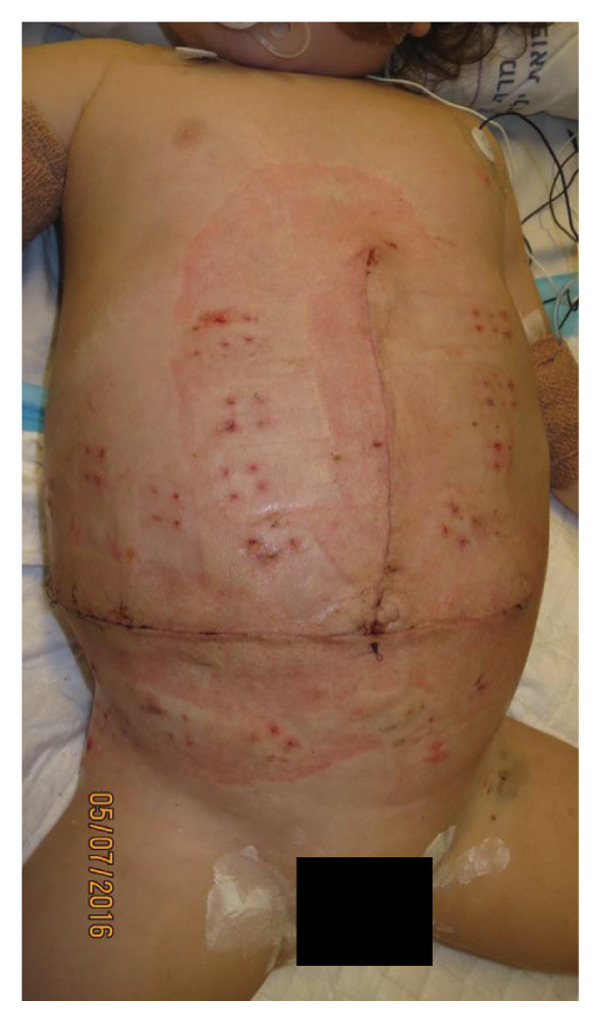
(g)

**Figure:**
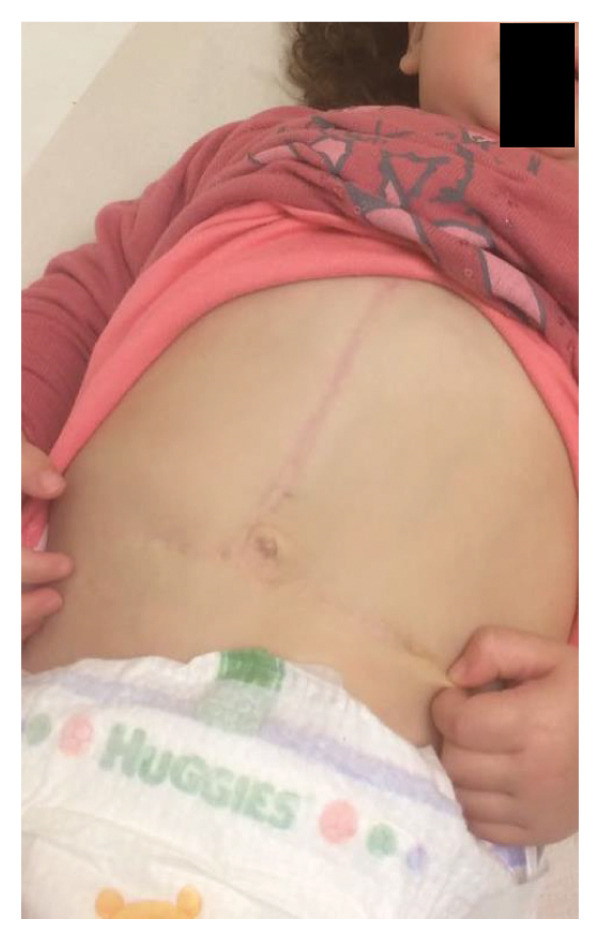
(h)

Four DGCT patients and two reference group patients underwent kidney transplants after LT. One reference group patient had a combined liver and pancreas transplant.

All five mortalities occurred in the reference group. The DGCT group had a median PICU stay of 17 days (IQR 14 to 21.5 days; range 9 to 122 days), significantly longer than the reference group of 8 days (IQR 6 to 11 days; range 0 to 82 days) (*p* < 0.001). The median hospital stay for the DGCT group was 35 days (IQR: 27.5 to 42 days; range 22 to 168 days), compared to 26 days (IQR 20.25 to 35 days, IQR 0 to 202 days) for the reference group (*p* = 0.003). When age, weight, and group were analyzed together using multivariable linear regression with log‐transformed outcomes, the DGCT group was independently associated with longer ICU stay (*β* = 0.72, 95% CI: 0.40–1.03, *p* < 0.001) and longer hospital stay (*β* = 0.28, 95% CI: 0.06–0.49, *p* = 0.014) after adjusting for age and weight.

The TRS remained in place for up to 4 weeks after DGCT implementation, enabling complete wound closure under reduced tension without dehiscence. Minimal skin injury from TRS staples healed with almost no visible scarring (Figures 1(g) and 1(h)), further improved by placing the TRS over a DAS sheet (Figures 1(f), 2(e), and 3(f)). No major bleeding, organ fistulization, or enteroatmospheric fistulas occurred following ROINPT treatment.

FIGURE 2Case 14: A 4‐month‐old infant (4.35 kg) with biliary atresia underwent LDLT (graft‐to‐recipient weight ratio 4.3%). Due to the high risk of intra‐abdominal pressure‐related complications, DGCT was planned preoperatively, avoiding immediate abdominal closure. (a) Primary fascial and skin closure was not feasible at the end of surgery. (b, c) A sterile fenestrated plastic bag was placed over the abdominal organs, with a soft open‐cell sponge (fenestrated lamination surface) and five TRS sets along the wound edges placed over a hydrocolloid dressing adhesive sheaths (HAS). The setup was sealed with a regulated, oxygen‐enriched irrigation and negative–pressure–assisted wound therapy dressing. (d) On POD 6, the patient underwent surgery for gastric outlet obstruction. Adhesions were lysed, and two‐thirds of the fascia were sutured. (e) After partial closure, the left lateral wound remained open, covered with a layered sponge dressing and four TRS sets. (f) On POD 10, septic shock prompted reoperation, revealing a bowel leak at the enteroenteric anastomosis, which was repaired. A repeated open abdomen approach was implemented.

**Figure:**
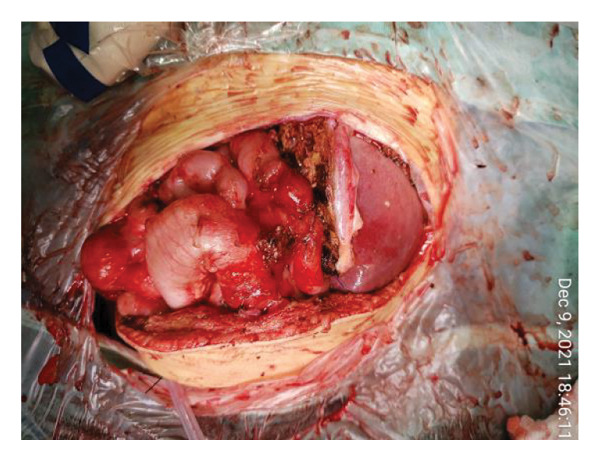
(a)

**Figure:**
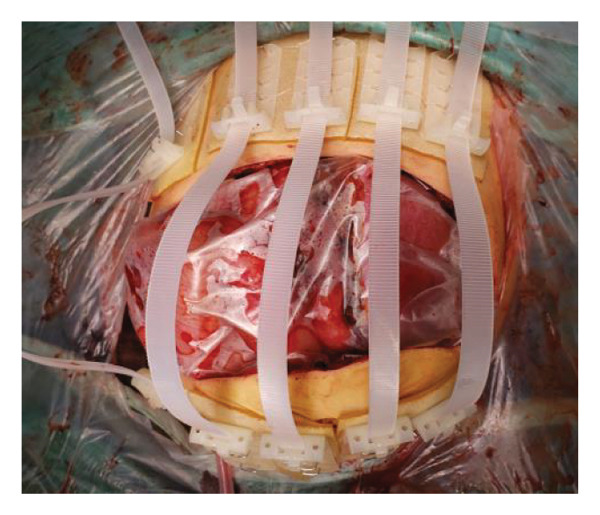
(b)

**Figure:**
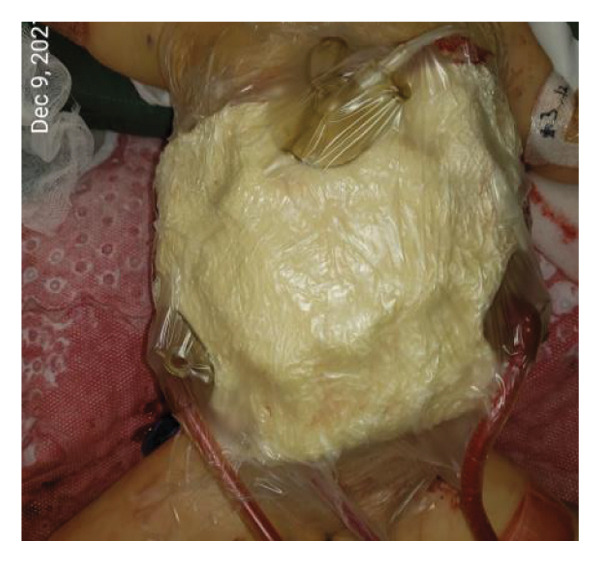
(c)

**Figure:**
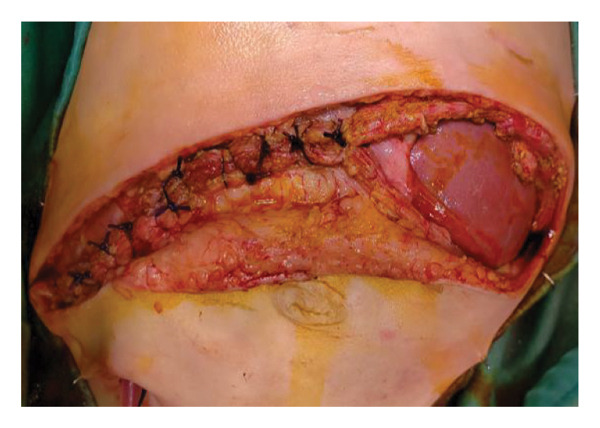
(d)

**Figure:**
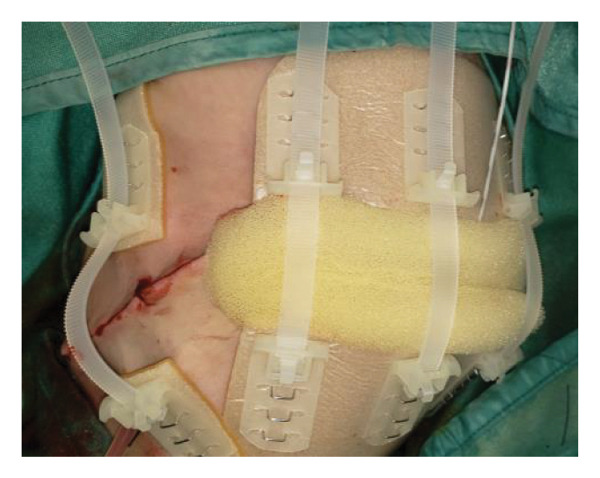
(e)

**Figure:**
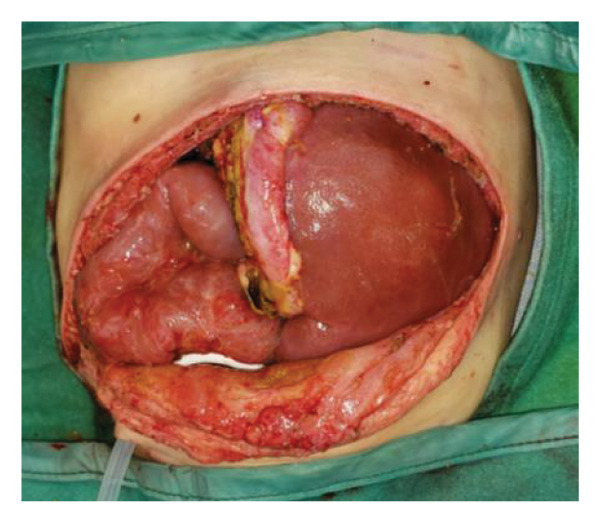
(f)

FIGURE 3Case 14 (Continued): (a) On POD 13, persistent gastric dilatation affecting ventilation necessitated Heineke–Mikulicz pyloroplasty. A Meckel’s diverticulum with imminent perforation was resected. (b) At surgery completion, DGCT was reapplied, covering the intestines with a fenestrated plastic sheath and approximating the wound edges with five TRS sets over hydrocolloid dressing adhesive sheaths. (c) The open abdomen was covered with ROINPT dressings. (d, e) On POD 18, primary fascial and skin closure was achieved. (f) On POD 24, TRS and ROINPT were removed, revealing complete wound closure with intact skin and excellent functional and aesthetic outcomes despite multiple procedures and wound reopening.

**Figure:**
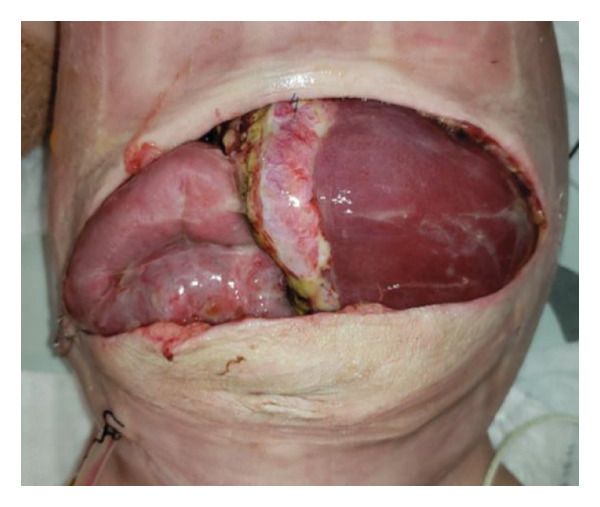
(a)

**Figure:**
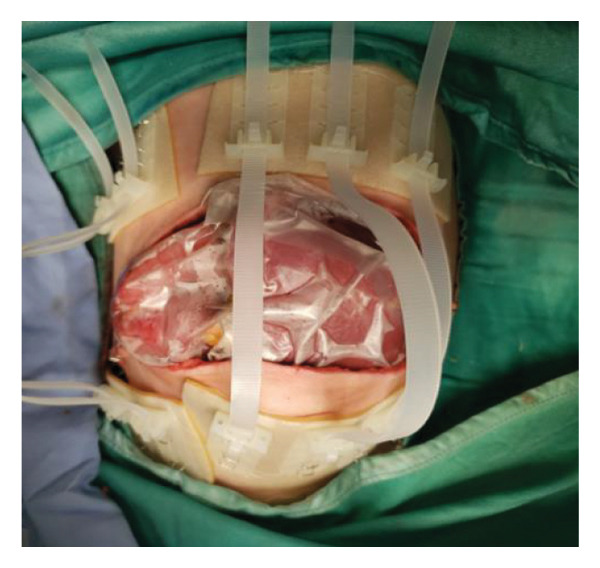
(b)

**Figure:**
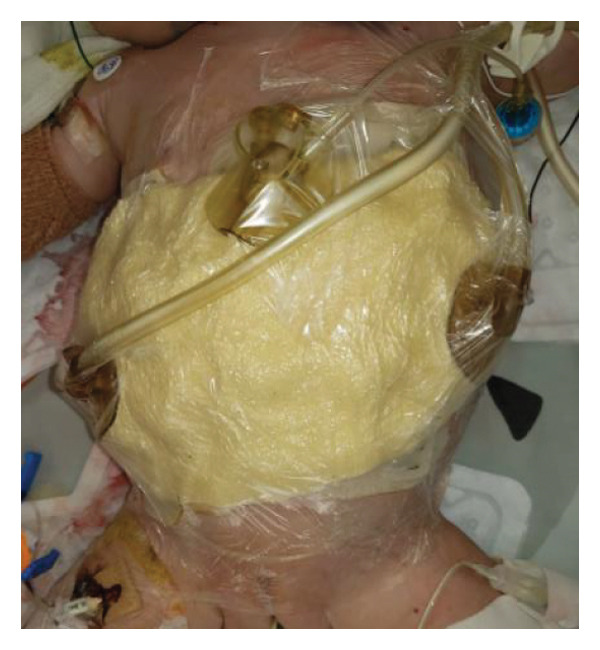
(c)

**Figure:**
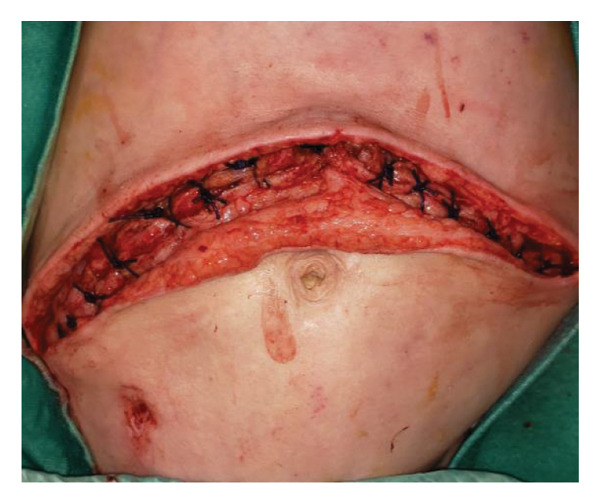
(d)

**Figure:**
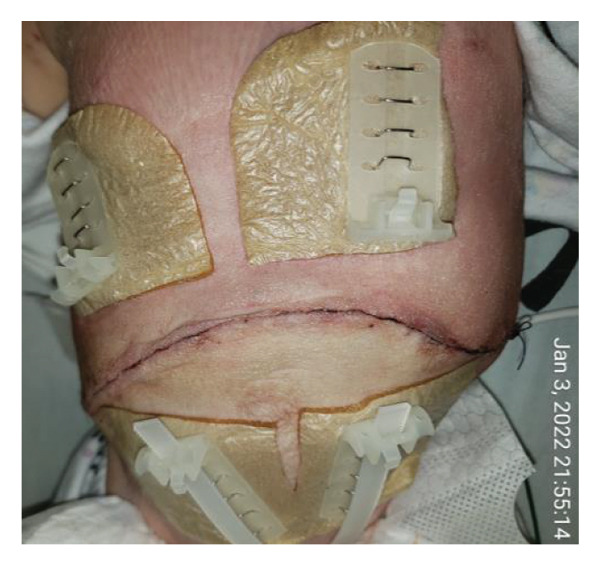
(e)

**Figure:**
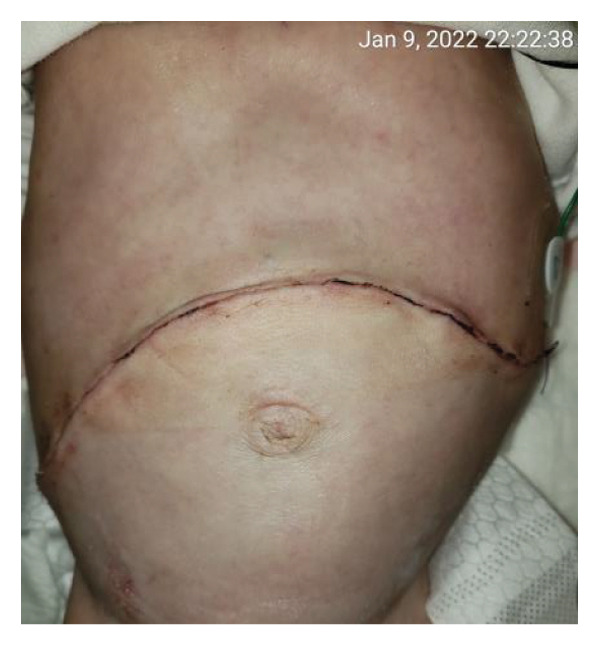
(f)

#### 3.5.1. Representative Cases

The following representative cases illustrate the application of the DGCT in managing OA. Procedural steps and clinical outcomes are presented in a visual format.

##### 3.5.1.1. Representative Case 1 (Figures 1(a), 1(b), 1(c), 1(d), 1(e), 1(f), 1(h))

The first clinical application of the DGCT was performed in a 1‐year and 7‐month‐old female with biliary atresia who underwent LDLT with a GRWR of 3.8%. At the conclusion of the procedure, due to excessive graft volume and bowel distension, primary abdominal closure was not achievable. A temporary abdominal closure was established using a Bogota bag, Kerlix® dressing, and sterile drape (Figure 1(a)).

On postoperative day (POD) 2, three TopClosure® sets were applied bedside to initiate gradual approximation and prevent muscular retraction (Figure 1(b)). A Doppler ultrasound‐guided approximation session was conducted in the PICU, allowing real‐time assessment of hepatic vascular flow during approximation (Figure 1(c)).

On POD 4, six TRS sets were applied in the operating room under Doppler guidance to further facilitate progressive closure, with the Bogota bag now replaced by Vcare α® vacuum dressings to provide a sealed, oxygen‐enriched negative pressure environment (Figure 1(d)). Despite continued closure efforts, reoperation was required on POD 8 due to fever and suspected intra‐abdominal pathology. Intraoperative findings revealed persistent fascial defects that precluded definitive closure. A Permacol™ biologic prosthesis was implanted to bridge midline and transverse fascial gaps, and full‐thickness skin closure was performed. TRS and ROINPT were reapplied to reduce tension, support tissue integration, and provide an enhanced environment for wound healing (Figures 1(e) and 1(f)).

By POD 14, all external devices were removed without complication (Figure 1(g)). At six months postoperatively, the patient developed a localized seroma at the incisional site, necessitating drainage and removal of the Permacol™ prosthesis. Importantly, no infectious complications occurred during the perioperative course. Liver graft function remained stable, and the patient achieved an excellent functional recovery with favorable cosmetic results (Figure 1(h)).

##### 3.5.1.2. Representative Case 14 (Figures 2(a), 2(b), 2(c), 2(d), 2(e), 2(f), 3(a), 3(b), 3(c), 3(d), 3(e), 3(f))

Case 14 represents a complex example of DGCT use in a small infant with high GRWR and bowel leak, requiring staged interventions while preserving the abdominal domain. A 5‐month‐old female infant with biliary atresia, weighing 4.35 kg, underwent LDLT with a GRWR of 4.3%. Given the anticipated graft–recipient size mismatch and the high risk of IAP‐related complications, DGCT was preoperatively planned. As expected, primary fascial and skin closure was not feasible at the end of the procedure (Figure 3(a)). A sterile fenestrated plastic sheath was placed over the abdominal contents, followed by the application of a fenestrated open‐cell PU sponge and five TRS sets anchored over HAS (Figure 3(b)). The configuration was sealed using a ROINPT system (Figure 3(c)).

On POD 6, the patient was reoperated for gastric outlet obstruction caused by adhesions, which were lysed, allowing partial fascial closure (Figure 3(d)). The residual lateral defect was managed with four additional TRS sets and a layered sponge dressing (Figure 3(e)). On POD 10, the patient developed septic shock. Emergency surgery revealed a bowel leak adjacent to the enteroenteric anastomosis, which was repaired (Figure 3(f)).

Subsequently, on POD 13, Heineke–Mikulicz pyloroplasty was performed due to ongoing gastric dilatation interfering with ventilation, and a Meckel’s diverticulum at imminent risk of perforation was resected (Figure 2(a)). DGCT was reapplied with five TRS sets and a new fenestrated nylon sheath (Figure 2(b)), followed by ROINPT coverage (Figure 2(c)). On POD 18, successful primary fascial and skin closure was achieved (Figures 2(d), 2(e)), and by POD 24, TRS and ROINPT were removed, revealing complete wound healing with excellent functional and aesthetic results (Figure 2(f)), despite multiple reoperations and wound reopening.

## 4. Discussion

### 4.1. Clinical Context and Rationale

The scarcity of size‐matched deceased liver donors for pediatric recipients has necessitated broader use of LDLT as a safe and effective alternative. The LLS is commonly used in pediatric LDLT; however, in smaller recipients, even reduced or hyper‐reduced LLS grafts may remain relatively oversized, increasing the risk of excessive closure tension, vascular compromise, and abdominal compartment physiology [16]. In such settings, delayed abdominal closure may be required in up to 30%–40% of cases and may increase the risk of wound infection, dehiscence, and incisional hernia [17, 18]. Previous studies have shown that LFS grafts can be transplanted safely when a strategy for delayed abdominal wall closure is anticipated [19]. Given the physiologic fragility and heightened infection risk in small infants undergoing LT, prosthesis‐sparing approaches are desirable. Our findings suggest that DGCT may provide a safe and effective alternative for achieving early delayed abdominal closure without permanent prosthetic materials.

### 4.2. OA Indications, DGCT Outcomes, Complications, and Predictive Indicators

In all 21 patients, the indication for OA management was either excessive closure tension or vascular compromise, with impaired arterial or portal inflow documented in 10 cases, most commonly in the setting of LFS graft mismatch. Overall, OA was required in 21 patients (16.3%) of our pediatric LT cohort because PC could not be achieved safely intraoperatively and/or because early postoperative abdominal compartment physiology or infection necessitated decompression and delayed closure. This incidence is lower than, but comparable to, previously reported pediatric LT series in which temporary abdominal closure or delayed sequential closure was required in up to 26.5% of recipients, particularly in LFS settings [20, 21].

These findings reinforce that OA should be regarded as an established component of perioperative management in selected pediatric LT recipients, particularly small infants and LFS transplant scenarios, rather than as a complication of DGCT. In this cohort, 90.5% of patients had a GRWR ≥ 2.5%, and 57.1% had a GRWR ≥ 3.5%, underscoring that limited abdominal domain and graft size mismatch are central predictors of OA requirement. Younger age and lower body weight were also significantly associated with OA use, consistent with reduced abdominal compliance and narrower physiologic reserve in smaller recipients.

DGCT should therefore be understood as a physiologically guided closure strategy within OA management. Using objective Doppler and IAP thresholds, DGCT enabled early delayed definitive closure in all 21 patients, with a median closure time of 8 days, while avoiding permanent prosthetic materials. Prosthetic reinforcement was required only temporarily in the earliest three cases during the learning curve, and no long‐term implantable prosthesis remained.

The clinically relevant postoperative complications in this cohort were biliary/intestinal leaks, sepsis, and the need for reoperation. Specifically, three of 21 patients (14%) developed biliary or intestinal leaks after DGCT application; one of these three patients developed sepsis and was successfully treated. Six reoperations (29%) were required, primarily for source control and/or staged completion of delayed primary fascial closure. Although this complication burden reflects the high‐risk nature of OA pediatric LT recipients, many with marked LFS mismatch, published pediatric LT cohorts managed with OA or delayed closure frequently report overall complication rates exceeding 30%–40%, particularly in the context of oversized grafts and reoperations [13, 21]. Accordingly, our safety outcomes appear at least comparable despite a high‐risk case mix (median GRWR 3.7%), while offering the additional advantage of achieving closure without permanent implantable materials in immunosuppressed infants.

### 4.3. Insights From Representative Cases

Case 1, our initial clinical application of DGCT, demonstrated the feasibility of controlled staged closure in a critically ill pediatric patient with marked graft–recipient mismatch (Figure 1). Real‐time Doppler monitoring allowed dynamic modulation of closure tension while preserving hepatic perfusion. Although a Permacol™ prosthesis was required to bridge a residual fascial defect, its later removal without complication illustrated the potential of DGCT to reduce long‐term prosthetic dependence. This early case informed the refinement of the technique in subsequent patients.

Case 14 further illustrates the adaptability of DGCT in a highly complex infant with high GRWR, postoperative bowel leak, and associated gastric and bowel complications. Despite multiple staged reoperations and repeated OA management, definitive fascial and skin closure was ultimately achieved without long‐term prosthetic use (Figures 2 and 3). Together, these representative cases demonstrate the ability of DGCT to preserve the abdominal domain, maintain graft viability, and support safe closure despite major perioperative complexity.

### 4.4. Advantages of DGCT Over Existing Techniques

Across the cohort, no graft failures, retransplantations, or DGCT‐related mortalities were observed. Late reoperation was necessary in only one case (Case 1) for the removal of a previously placed permanent implant. As our experience has accumulated, we have gained greater confidence in using OA proactively when clinically indicated, rather than forcing closure in the setting of excessive tension or vascular compromise.

DGCT offers several advantages over conventional delayed‐closure techniques. The elasticity of the abdominal wall permits effective exploitation of the skin’s viscoelastic properties through TRS‐mediated mechanical creep, enabling gradual external tissue stretching and progressive whole‐thickness approximation of the cutaneous, muscular, and fascial layers. This supports a prosthesis‐sparing strategy and, in our view, eliminates the rationale for internal tissue expanders in this setting. In addition, by distributing tensile forces over a broader surface area, typically via a hydrocolloid interface, away from the wound edges, the TRS may mitigate edge ischemia and thereby reduce the risks of necrosis, wound dehiscence, local skin injury, and related complications [14, 22].

### 4.5. Comparative Applications and Literature Support

Figures 1, 2, and 3 illustrate the technical and clinical consequences of effective tension reduction. Although sutures contribute to wound approximation, their small cross‐sectional area and rigid composition concentrate mechanical stress at points of contact, underscoring the importance of the TRS in managing high‐tension wounds [22]. Previous studies have shown TopClosure® to be a viable alternative to skin grafts, flaps, and tissue expanders in selected settings [14, 22]. In adults, large OA defects have also been closed early, primarily via stress relaxation using tension sutures over TRS sets, without permanent prosthetic implants [23].

In pediatric LT, however, early fascial closure can only be pursued safely under strict physiologic monitoring. Excessive tension on the fascia and skin, as well as the use of conventional high negative pressure vacuum dressings (e.g., 125 mmHg), may compromise graft perfusion. In this context, TRS‐mediated staged approximation under repeated Doppler assessment provides a reversible and controlled means of progressing toward closure, as illustrated in Figures 1(a), 1(b), 1(d), and 1(e) and in the Supporting video (File S1).

### 4.6. Technical Features of ROINPT

NPWT is a well‐established modality for managing complex wounds, including abdominal closure in pediatric LT [24]. However, the ROINPT technique integrated with the Vcare α® system offers several features that may be particularly advantageous in this setting. It uses a specialized off‐white, soft, open‐cell PU sponge that facilitates early recognition of local complications such as bleeding or infection through visible color changes.

When the fenestrated laminated sponge is placed over a thin fenestrated plastic sheet covering the abdominal contents, the system facilitates evacuation of peritoneal fluid, reduces edema and contaminant burden, and protects the underlying viscera. The vacuum operates within a controlled range of 40–60 mmHg, which may reduce the risks of bowel erosion, perforation, bleeding, vascular compromise, and excessive topical pressure on intra‐abdominal structures [15]. Higher suction levels are deliberately avoided to prevent granulation overgrowth, cocoon formation, and intra‐abdominal adhesions that may hinder delayed closure.

Early use of TRS in combination with low‐regulated vacuum also helps prevent skin and fascial retraction, reduces wound gap progression, and allows bedside approximation between operations without repeated wound exposure. In addition, conventional vacuum systems may lower atmospheric pO_2_ and thereby promote anaerobic growth, whereas ROINPT increases local atmospheric pO_2_ to 60%–80%, suppressing anaerobic bacterial proliferation [15]. The sealed system also provides wound insulation, controlled drainage, continuous irrigation, and hydrodynamic cleansing, thereby enhancing decontamination [25]. Monitoring the daily fluid volume in the collection container may also assist in fluid‐balance assessment.

Accordingly, the primary functions of ROINPT in this setting are wound insulation, decontamination, localized oxygen enrichment, and decongestion, rather than stimulation of granulation tissue growth. Dressing changes are typically performed every 3–5 days, without increasing the risk of wound infection or sepsis, thereby reducing workload and patient discomfort while supporting safer and potentially faster closure.

### 4.7. Wound Healing and Broader Applications

The DGCT framework, integrating TRS and ROINPT, provides flexibility in complex surgical situations because these components may be used separately or in combination with temporary adjuncts such as Bogota bags or surgical mesh when clinically necessary. Early integration of DGCT into operative planning may reduce reliance on permanent prosthetic aids, streamline OA management, limit fascial lateralization, and accelerate progression toward closure. When postoperative complications necessitate renewed OA management, the same framework can be reapplied to restore controlled, staged closure.

Related applications in trauma and adult OA management support the broader technical versatility of the underlying approach [23, 26–28]. In the present cohort, these principles translated into effective early abdominal closure under highly challenging pediatric transplant conditions.

### 4.8. Limitations

This study has several important limitations. It is a retrospective, single‐center observational cohort and was not designed to provide formal causal inference or definitive comparative effectiveness analysis. The absence of a true control group limits interpretation of between‐group comparisons, particularly because patients requiring OA were younger and smaller and therefore inherently at higher physiologic risk.

In addition, both TRS and ROINPT require a learning curve, and meticulous application is essential in pediatric patients to avoid excessive tension, impaired perfusion, or compartment physiology. Gradual closure is particularly important when wound edges and fascia cannot be approximated safely in the setting of LFS grafts or adverse local conditions. Continuous physiologic monitoring remains essential during staged closure, particularly with respect to Doppler perfusion and IAP.

Our early experience also indicates that when uncertainty arises, maintaining OA under controlled DGCT conditions is safer than forcing closure. For this reason, initial application in adult patients may serve as a useful preparatory stage before adoption in pediatric LT. We also strongly encourage expert consultation during early institutional implementation. Finally, although the present results are encouraging, larger prospective studies are needed to validate these findings and to better define the broader applicability of this strategy.

## 5. Conclusion

The DGCT approach, combining TRS technology and ROINPT under real‐time Doppler ultrasound guidance, enabled early and safe abdominal closure in all 21 pediatric liver transplant recipients in this series, most of whom received LFS grafts associated with a high risk of excessive closure tension and vascular compromise. PC was achieved within a median of 8 days without the need for permanent prosthetic materials or graft size reduction. No patients experienced graft failure, retransplantation, or mortality. By enabling reversible, physiologically guided tension modulation and optimization of the local wound environment, DGCT appears to be a feasible prosthesis‐sparing strategy for selected high‐risk pediatric liver transplant recipients requiring OA management. In light of prior published experience in LFS graft management and the broader cumulative experience with this approach in complex OA settings, these findings suggest potential relevance beyond this cohort; however, prospective validation in larger comparative studies is required before definitive conclusions regarding donor‐pool expansion or broader applicability can be drawn.

**Nomenclature**
DGCT: Topaz–Gurevich Doppler‐guided controlled‐closure technique
DDLT: Deceased donor liver transplantation
GRWR: Graft‐to‐recipient weight ratio
IAP: Intra‐abdominal pressure
HAS: Hydrocolloid adhesive sheath
LFSLT: Large‐for‐size liver transplantation
LLS: Left lateral segment
LFS: Large‐for‐size
LDLT: Living donor liver transplantation
NPWT: Negative pressure wound therapy
OA: Open abdomen
PC: Primary closure
PICU: Pediatric intensive care unit
PTFE: Polytetrafluoroethylene
ROINPT: Regulated, oxygen‐enriched irrigation and negative pressure–assisted wound therapy
SCMC: Schneider Children’s Medical Center
TRS: Tension relief system

## Author Contributions

Moris Topaz, MD, PhD, and Michael Gurevich, MD, conceived the study and led development and clinical implementation of the DGCT approach. Moris Topaz, MD, PhD; Michael Gurevich, MD; and Tomer Mendelson, MD, contributed to study design, data interpretation, and manuscript drafting. Tomer Mendelson, MD, coordinated data extraction and database assembly. Haya Fischer, MD, performed clinical data verification and assisted with data curation and manuscript preparation. Elhanan Nahum, MD, and Avichai Weissbach, MD, contributed to perioperative and PICU management data collection and interpretation. Yael Glassberg Mozer, MD, and Orith Waisbourd‐Zinman, MD, contributed to hepatology/transplant indication data interpretation and critical manuscript revision. Sigal Aizner, MD, and Eviatar Nesher, MD, contributed to transplant surgical data interpretation and critical revision of the manuscript for important intellectual content.

All authors contributed significantly to the conception, design, drafting, and critical revision of the manuscript. Each author played a substantial role in ensuring the scientific rigor and clinical relevance of the study. Their combined expertise in pediatric liver transplantation, surgical techniques, and postoperative management has been instrumental in shaping the findings and conclusions presented.

## Funding

No external funding was received for this study.

## Disclosure

All authors critically reviewed the manuscript, approved the final version, and agreed to be accountable for all aspects of the work.

We state that IVT Medical Ltd. had no role in the study design; collection, analysis, or interpretation of data; writing of the manuscript; or the decision to submit the manuscript for publication

## Conflicts of Interest

Dr. Moris Topaz is the developer and patent holder of the TopClosure® Tension Relief System, Vcare α®, and MeCare® systems and serves as CEO and a shareholder of IVT Medical Ltd., the manufacturer of these devices. The other authors declare no conflicts of interest.

## Supporting Information

Additional supporting information can be found online in the Supporting Information section.

## Supporting information


**Supporting Information** A Supporting video file is provided separately to illustrate the basic surgical approach, as detailed in Supporting Information (File S1).

## Data Availability

The data that support the findings of this study are available upon request from the corresponding author. The data are not publicly available due to privacy or ethical restrictions.
